# Identification of a recessive gene *RgM4G52* conferring red glume, stem, and rachis in a *Triticum boeoticum* mutant

**DOI:** 10.3389/fpls.2024.1459505

**Published:** 2024-08-26

**Authors:** Longyu Chen, Junqing Zhang, Pan Ma, Yongping Miao, Lei Wu, Ke Zhou, Jiaru Yang, Minghu Zhang, Xin Liu, Bo Jiang, Ming Hao, Lin Huang, Shunzong Ning, Xuejiao Chen, Xue Chen, Dengcai Liu, Hongshen Wan, Lianquan Zhang

**Affiliations:** ^1^ State Key Laboratory of Crop Gene Exploration and Utilization in Southwest China, Sichuan Agricultural University, Chengdu, China; ^2^ Triticeae Research Institute, Sichuan Agricultural University, Chengdu, China; ^3^ Key Laboratory of Wheat Biology and Genetic Improvement on Southwestern China (Ministry of Agriculture and Rural Affairs), Crop Research Institute, Sichuan Academy of Agricultural Sciences, Chengdu, China; ^4^ Environment-Friendly Crop Germplasm Innovation and Genetic Improvement Key Laboratory of Sichuan Province, Crop Research Institute, Sichuan Academy of Agricultural Sciences, Chengdu, China

**Keywords:** *Triticum boeoticum*, synthetic amphiploid, red glume, gene mapping, recessive gene, BSE-Seq

## Abstract

Anthocyanins are plant secondary metabolites belonging to the polyphenol class of natural water-soluble phytopigments. The accumulation of anthocyanins in different plant tissues can improve plant survival under adverse conditions. In addition, plants with the resulting colorful morphology can be utilized as landscape plants. *Triticum boeoticum* (syn. *Triticum monococcum* ssp. *aegilopoides*, 2n=2x=14, A^b^A^b^) serves as a valuable genetic resource for the improvement of its close relative common wheat in terms of enhancing resilience to various biotic and abiotic stresses. In our previous study, the EMS-mutagenized mutant Z2921 with a red glume, stem, and rachis was generated from *T. boeoticum* G52, which has a green glume, stem, and rachis. In this study, the F_1_, F_2_, and F_2:3_ generations of a cross between mutant-type Z2921 and wild-type G52 were developed. A single recessive gene, tentatively designated *RgM4G52*, was identified in Z2921 via genetic analysis. Using bulked segregant exome capture sequencing (BSE-Seq) analysis, *RgM4G52* was mapped to chromosome 6AL and was flanked by the markers *KASP-58* and *KASP-26* within a 3.40-cM genetic interval corresponding to 1.71-Mb and 1.61-Mb physical regions in the Chinese Spring (IWGSC RefSeq v1.1) and *Triticum boeoticum* (TA299) reference genomes, respectively, in which seven and four genes related to anthocyanin synthesis development were annotated. Unlike previously reported color morphology-related genes, *RgM4G52* is a recessive gene that can simultaneously control the color of glumes, stems, and rachis in wild einkorn. In addition, a synthetic *Triticum dicoccum*–*T. boeoticum* amphiploid Syn-ABA^b^-34, derived from the colchicine treatment of F_1_ hybrids between tetraploid wheat PI 352367 (*T. dicoccum*, AABB) and Z2921, expressed the red stems of Z2921. The flanking markers of *RgM4G52* developed in this study could be useful for developing additional common wheat lines with red stems, laying the foundation for marker-assisted breeding and the fine mapping of *RgM4G52*.

## Introduction

Anthocyanins are flavonoid pigments that are important for plant adaptation to biotic and abiotic stress conditions (Laikova et al., 2005). In common bread wheat (*Triticum aestivum* L.), pigmentation caused by anthocyanins can occur on leaves, stems, auricles, glumes, pericarp aleurone, coleoptiles, and anthers. Pigment accumulation in crop plants can not only be used as a morphological marker to assist in breeding and research related to impurity removal, gene functions, pigment synthesis pathways, photosynthesis, and other related theories but also provide agroecological tourism value for humans (Song et al., 2020). The anthocyanin pigmentation of different parts of plants is related to their adaptation to environmental stress conditions. In wheat, purple coleoptiles, stems, and anthers are reportedly related to resistance to bunt (Bogdanova et al., 2002). It was shown that the lines with dark-purple grains and coleoptiles demonstrated a higher seedling drought tolerance than plants with uncolored pericarp and light purple coleoptiles (Shoeva et al., 2017). The relationship between accumulation of anthocyanins in wheat coleoptiles and cold treatment has been shown (Gordeeva et al., 2013). Furthermore, the purple-grained NILs had better viability after accelerated aging compared with the recurrent parent lacking anthocyanins (Gordeeva and Khlestkina, 2013). The knowledge about specific features of anthocyanin biosynthesis regulation in wheat can be useful for improvement of its adaptation to biotic and abiotic stress conditions. In addition, anthocyanins are important for maintaining human health and preventing cardiovascular diseases, carcinogenesis, inflammation, and many other human pathological conditions (Lila, 2004).

Anthocyanin accumulation occurs naturally in some species. However, it has also been reported that some changes in pigment accumulation occur in mutant plant populations induced by chemical mutagens. Chemical mutagens, among which ethyl methane sulfonate (EMS) is the most widely used and effective in crop mutagenesis breeding, can induce plants to produce heritable mutants (Li et al., 2017; Greene et al., 2003; Henry et al., 2014). There is a high frequency of EMS-induced point mutagenesis, primarily involving G–C to A–T conversion, with relatively few chromosomal aberrations. Most of the mutations are easy to screen (Greco et al., 2001) and can be used to induce variation in plants, construct mutant libraries, and provide rich basic genetic material for the study of plant gene function. Liu et al. (2009) obtained a rice (*Oryza sativa* L.) *YGL4* mutant with a yellow–green leaf color through the EMS mutagenesis of Jinhui 10 cultivar. EMS-induced mutant germplasm can be effectively used to mine new genes, facilitate functional genomics research, and accelerate breeding programs (Hohmann et al., 2005). Mutagenetic breeding techniques make it possible to overcome the disadvantages of the long conventional breeding cycle, which is slow and comes with difficulties in producing mutation variation. Moreover, mutagenic breeding techniques can bring about breakthroughs in the creation of new crop cultivars, germplasms, and genetic materials and can enable some special problems in breeding work to be solved.

*Triticum boeoticum* (Syn. *Triticum monococcum* ssp. *aegilopoides*, 2n=2x=14, A^b^A^b^), the wild progenitor of *Triticum monococcum* ssp. *monococcum* (2n=2x=14, A^m^A^m^), represents a valuable genetic resource for improving the ability of its close relative common wheat in terms of coping with various biotic and abiotic stresses (Budak et al., 2013; Ahmed et al., 2023; Wang et al., 2023). In addition, *T. boeoticum* is one of the sources of the blue grain trait controlled by blue aleurone layer 2 (*Ba2*) (Zeller et al., 1991; Zeven, 1991; Dubcovsky et al., 1996; Singh et al., 2007; Yu et al., 2017; Liu et al., 2021). To date, the salt tolerance genes *Nax2* and *Nax1*; the powdery mildew resistance genes *Pm25*, *PmTb7A.1*, and *PmTb7A.2*; the leaf rust resistance gene *Sr22*; the stripe rust resistance locus *QYrtb.pau-5A*; and the stripe rust resistance gene *YrZ15-1370* have been successfully introduced into common wheat (Paull et al., 1994; Shi et al., 1998; Chhuneja et al., 2008; Munns et al., 2012; Tounsi et al., 2016; Elkot et al., 2015; Zhang et al., 2021).

Synthetic amphiploids play an important role in wheat breeding and evolutionary studies (Gill et al., 1988; Megyeri et al., 2011; Ahmed et al., 2014; Badaeva et al., 2016; Li et al., 2018; Liu et al., 2022, 2023). Many studies have aimed to generate amphidiploids from hybrids of wheat with related species from the genera *Aegilops*, *Secale*, *Thinopyrum*, and *Triticum* (Nemeth et al., 2015). Recently, 18 synthetic *Triticum turgidum*–*Triticum boeoticum* amphiploids were identified using cytological methods, and their nutritional compositions were evaluated (Liu et al., 2023). Artificial amphidiploids are regarded as a source of genetic variation for improving wheat (Kroupin et al., 2019).

Since the first mention of the expression of purple color traits in wheat, studies on the inheritance of these characteristics have made great progress in revealing the molecular-genetic mechanisms of anthocyanin pigment biosynthesis and its regulation in wheat (Shoeva and Khlestkina, 2015). In our previous study, a mutant, Z2921, with a red glume, stem, and rachis was produced from an EMS-mutagenized population of *T. boeoticum* G52 in M_4_ generation. In addition, a synthetic *T. dicoccum*–*T. boeoticum* amphiploid, Syn-ABA^b^-34, derived from the colchicine treatment of F_1_ hybrids of *T. dicoccum* PI 352367 and Z2921, expressed the red stem from Z2921. The objectives of the present study were (1) to identify and map the genes conferring resistance to the red glume, stem, and rachis in Z2921 by using bulked segregant exome capture sequencing (BSE-Seq) analysis and (2) to characterize the new synthetic *T. dicoccum*–*T. boeoticum* amphiploid Syn-ABA^b^-34 via cytological methods.

## Materials and methods

### Plant materials

*T. boeoticum* G52, the Z2921 mutant, *T. dicoccum* PI352367, and the synthetic *T. dicoccum*–*T. boeoticum* amphiploid Syn-ABA^b^-34 were used in this study. The M_4_ generation of Z2921, which originated from the 0.4% EMS treatment of 3,000 seeds of the *T. boeoticum* accession G52, was used. Syn-ABA^b^-34 was derived from the colchicine treatment of F_1_ hybrids from PI 352367×Z2921. G52 was kindly provided by George Fedak at the Ottawa Research and Development Centre for Agriculture and Agri-Food (Ottawa, ON, Canada) in 2012. PI 352367 was kindly provided by the USDA-ARS germplasm bank (http://www.ars-grin.gov). All materials used in this study were kept at the Triticeae Research Institute of Sichuan Agricultural University.

### Cytological observations

Sc-GISH was conducted using *T. boeoticum* genomic DNA as a probe according to previously published methods (Wang et al., 2019). Total genomic DNA from G52 was labeled with fluorescein-12-dUTP (Roche Diagnostics Australia, Castle Hill, NSW) using nick translation. The chromosomes were counterstained with 4′,6-diamidino-2-phenylindole (DAPI) and pseudocolored red. Hybridization signals were visualized and captured using an Olympus BX-63 epifluorescence microscope equipped with a Photometric SenSys DP70 CCD camera (Olympus, Tokyo, Japan). The raw images were processed using Photoshop v.7.1 (Adobe Systems Inc., San Jose, CA, USA).

Chromosome pairing observation in pollen mother cells (PMCs) was performed as described previously (Zhang et al., 2007). For meiotic analysis, at least 50 PMCs were observed for Z5471. Ring bivalents (ring II) and rod bivalents (rod II) were counted, and their average numbers were calculated.

### Phenotypic investigation

Field evaluations of glumes, stems, and rachides in G52, Z2921, PI 352367, Syn-ABA^b^-34, G52×Z2921 F_1_, and G52×Z2921 F_2_ individuals as well as their corresponding F_2:3_ families were performed at the experimental field of the Triticeae Research Institute, Sichuan Agricultural University, Wenjiang. The colors of the glumes, stems, and rachides were recorded as red or green from the jointing stage to the mature stage during the growth cycle. Each plant was 10 cm apart within rows and 30 cm apart between rows and was 1.5 m in length. The color phenotypes of all the materials in each generation were investigated.

### Bulked segregant exome capture sequencing

Genomic DNA was extracted via the CTAB method (Chatterjee et al., 2002). Phenotypically contrasting F_2:3_ families with different glume/stem/rachis colorations in the field were used to construct red and green glume/stem/rachis DNA pools for BSE-Seq. Equal amounts of DNA from 20 homozygous red-phenotype families and 20 homozygous green-phenotype families were pooled for bulked segregant exome capture sequencing (Ji et al., 2023). The DNA samples were subjected to exome capture sequencing, a technology developed by Chengdu Tcuni Technology (Chengdu, China). Sequence quality was controlled using Trimmomatic v0.36 software (Bolger et al., 2014). DNA reads of the wild-type and mutant bulks were aligned to the reference genome sequence of Chinese Spring v1.1 (IWGSC, 2018) using STARv2.5.1b software (Dobin et al., 2013). The unique and high-confidence alignments were applied to call SNP variants using GATK v3.6 software (McKenna et al., 2010). SNP variants with Fisher’s exact test (FET) *P* values < 1e^−8^ and allele frequency difference (AFD) >0.6 were considered associated with the red glume/stem/rachis phenotype and were then used as templates to develop SNP markers (Li et al., 2020).

### Kompetitive allele−specific PCR assays

The red-phenotype-related SNPs and the 500-bp flanking sequences were used to design the Kompetitive allele−specific PCR (KASP) primers and test polymorphisms in the parental lines and the wild-type and mutant DNA bulks. Polymorphic markers that could be reliably scored were genotyped in the F_2_ segregation population of G52×Z2921. For each KASP assay, a 10-µl reaction volume containing 5 µl of 2 KASP master mix (Biosearch Technologies), 1.4 µl of primer mix (a mixture of 0.168 µM each forward A1 and A2 primer and 0.42 µM of reverse primer), 100 ng of genomic DNA, and 2.6 µl of ddH_2_O was prepared. The CFX96 Touch™ real-time PCR detection system (Bio-Rad, USA) was used for amplification under the following conditions: 15 min at 94°C, 10 touchdown cycles of 20 s at 94°C, 60 s at 65°C–57°C (decreasing by 0.8°C per cycle), and 32 cycles of 20 s at 94°C, 60 s at 57°C.

### Data analysis

Chi-square (*χ^2^*) tests were used to determine the goodness of fit for the observed segregation and expected ratios of the F_2_ and F_2:3_ populations. Linkage analysis was performed using MAPMAKER/EXP v3.0b (Lander et al., 1987). The Kosambi function was used to convert recombination values to genetic distances (Kosambi, 1943). A logarithmic odds (LOD) ratio of 3.0 and a maximum distance of 50.0 cM were set as the thresholds for the declaration of linkage. The genetic linkage map was drawn using MapDraw v2.1 software (Liu and Meng, 2003).

### Candidate gene analysis

The corresponding sequences of the markers *KASP-58* and *KASP-26* linked to *RgM4G52* were subjected to BLAST searches against the genomes of common wheat cv. Chinese Spring v1.1 (Appels et al., 2018) and the genome of *Triticum boeoticum* TA299 (Ahmed et al., 2023). Gene annotations between the flanking markers of the two genomes were retrieved from the databases Ensembl Plants (http://plants.ensembl.org/index.html) and Swiss-Prot (http://www.gpm-aw.com/html/swi-ss-prot.html). Collinearity analysis was performed on the DEGs related to the function of anthocyanin synthesis among the parents and mixed pools.

## Results

### Genetic analysis of genes related to red glumes, stems, and rachides

**Figure 1:**
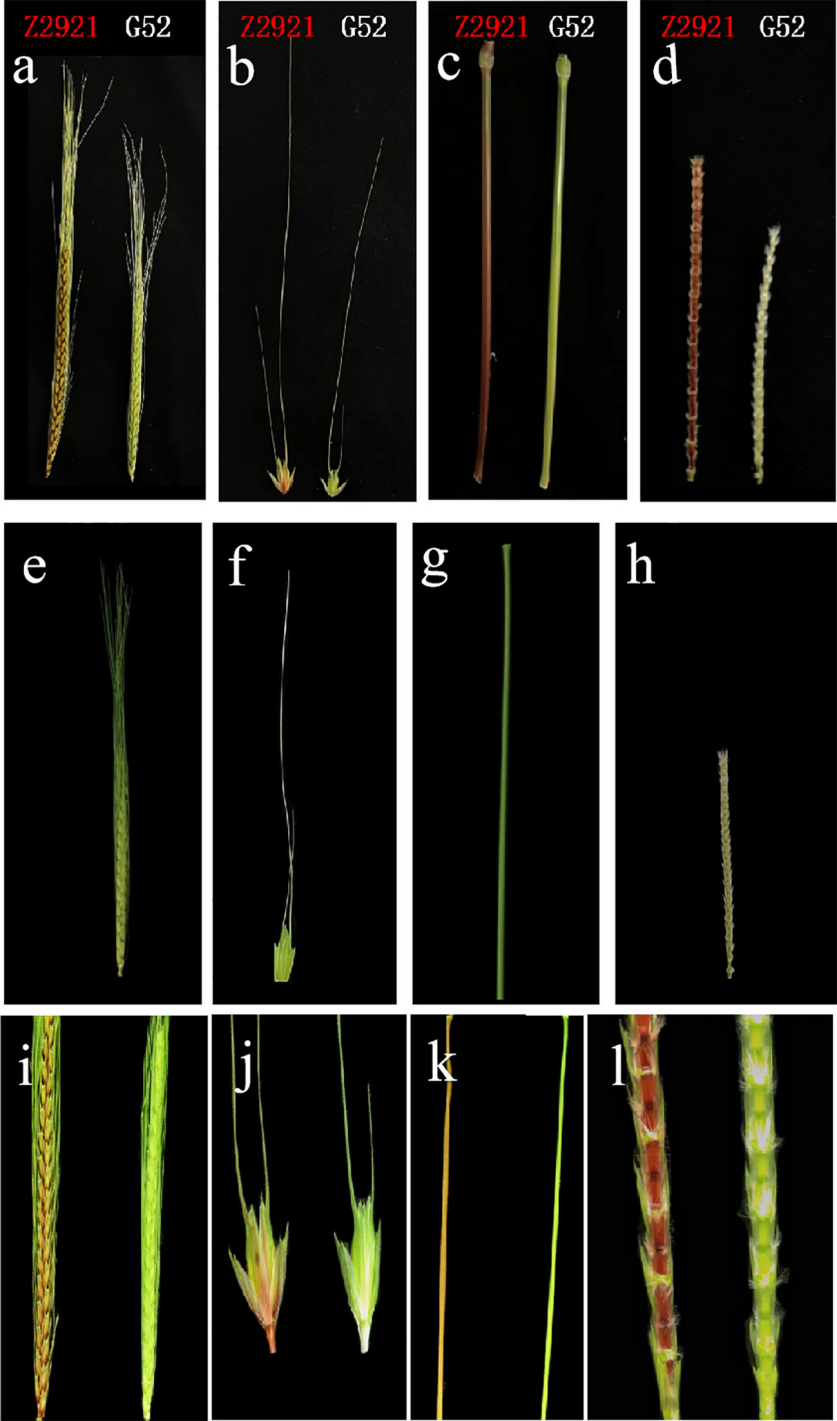
The coloration of spikes **(A)**, spikelets (glumes) **(B)**, stems **(C)** and rachides **(D)** of Z2921 and G52; The coloration of spikes **(E)**, spikelets (glumes) **(F)**, stems **(G)**, and rachides **(H)** of G52×Z2921 F_1;_ The coloration of spikes **(I)**, spikelets (glumes) **(J)**, stems **(K)**, and rachides **(L)** of G52×Z2921 F_2_ individual plants.

From the jointing stage to the mature stage, the EMS mutant Z2921 exhibited red glumes, stems and rachides (**Figures 1A–D**), and G52 exhibited green glumes, stems, and rachides. Z2921 was crossed with G52 to develop F_1_, F_2_, and F_2:3_ populations for genetic analysis of the genes conferring red glumes, stems, and rachides in Z2921. The glume, stem, and rachis coloration of all the F_1_ plants were similar to those of the parent G52 plants, with green glumes, stems, and rachides (**Figures 1E–H**). The F_2_ population segregated into 46 green-phenotype and 18 red-phenotype strains (**Figures 1I–L**), fitting a 1G:3R ratio (χ^2 =^ 0.333, p=0.564) (**Table 1**), indicating that the red phenotype was conferred by a single recessive gene, tentatively designated *RgM4G52*. The segregation rate of the F_2:3_ population composed of 64 families was 14 (homozygous green type):30 (heterozygous):17 (homozygous red type) (χ_1:2:1_^2 =^ 0.097, *p* = 0.953), which is consistent with the segregation results of the F_2_ population (**Table 1**).

**Table 1:** Genetic analysis of red genes in F_1_, F_2_, and F_2:3_ families of Z2921 × G52.

Parents and cross	Generation^a^	No. of plants/families	Observed ratio^b^			Actual ratio	Expected ratio	χ^2^	*P-value*
			G	Seg	R				
G52	Pg	10	10						
Z2921	P_R_	10			10				
Pg × P_R_	F_1_	10	10						
	F_2_	64	46		18	2.6:1	3:1	0.333	0.564
	F_2:3_	61	16	28	17	1:1.75:1.06	1:2:1	0.097	0.953

^a^P_S_, wild-type (green color) parent G52; P_Ss_, mutant type (red color) parent Z2921.

^b^R, homozygous red; Seg, segregating within F_2:3_ families; G, homozygous green.

### BSE-Seq analysis

**Figure 2:**
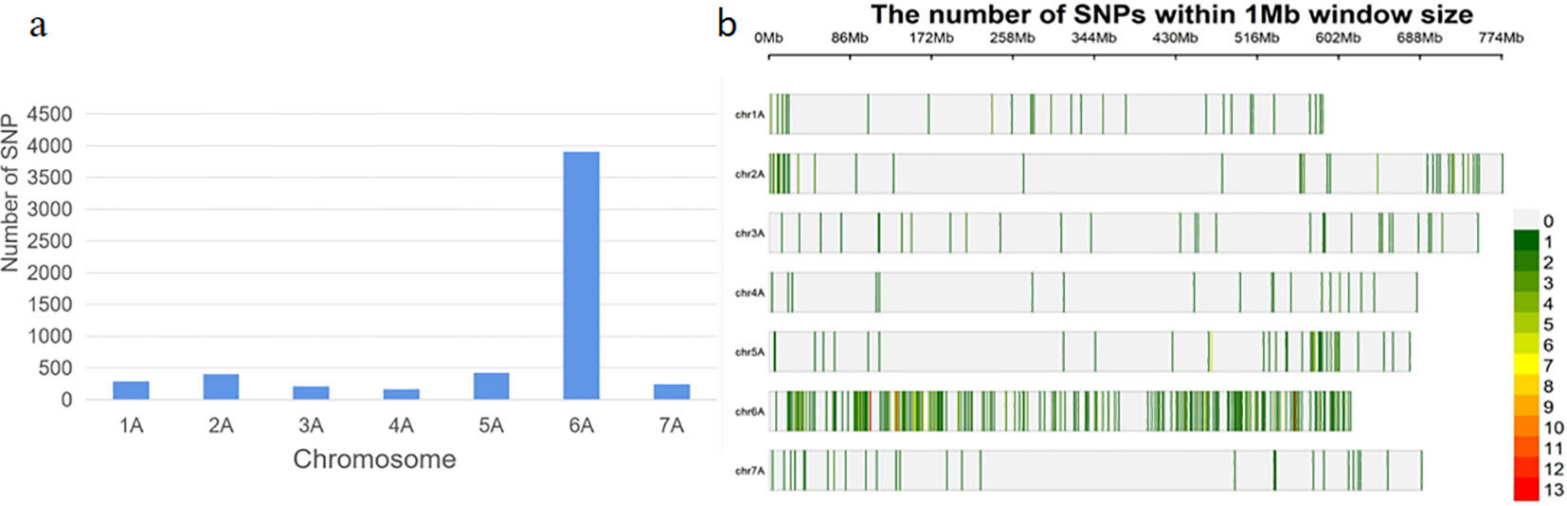
Distribution of SNPs from DNA bulks. **(A)** Number of SNPs (|AFD| > 0.6, *P* value <1e^−8^) distributed on different wheat chromosomes; **(B)** enrichment of SNPs within a 1-Mb window size on wheat chromosomes.

The DNA samples of the red bulk and the green bulk were subjected to BSE-Seq analysis, which generated 224,290,036 and 177,194,890 raw reads, respectively. After quality control, 224,266,906 and 177,176,312 high-quality reads from the red bulk and the green bulk, respectively, were uniquely mapped to the Chinese Spring genome (IWGSC RefSeq v1.1). A total of 5651 SNPs (*p*< 1^e-8^ and |AFD|>0.6) were identified from these reads using GATK v4.0 software (**Figure 2B**). A total of 348 SNPs were located within a 5-Mb genomic interval (555 Mb–560 Mb) on the long arm of chromosome 6A (**Figure 2A**) in the Chinese Spring reference genome; these SNPs were regarded as candidate SNPs linked to *RgM4G52*.

### Molecular mapping of *RgM4G52*

**Figure 3:**
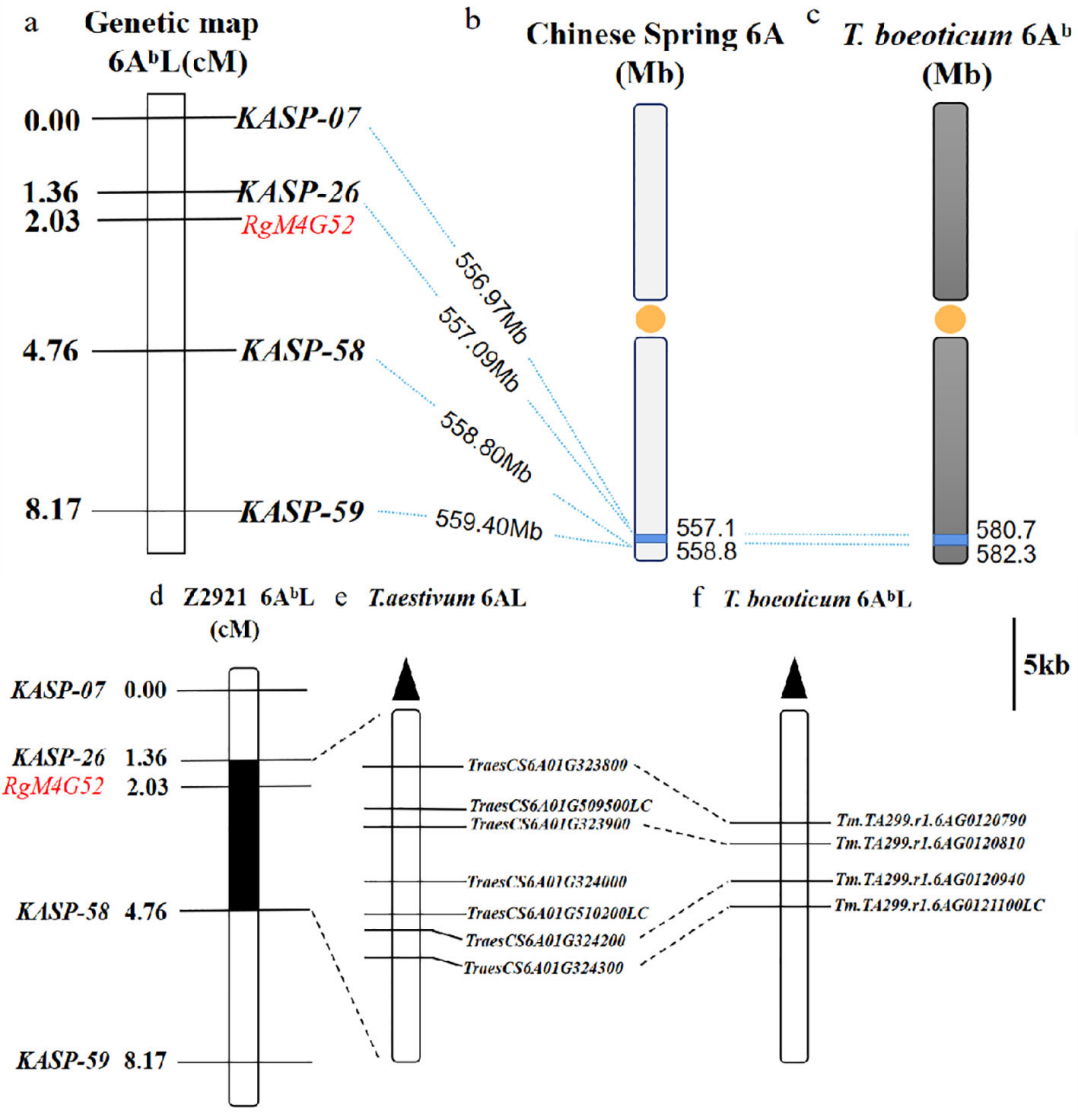
Genetic linkage map of the *RgM4G52* gene on chromosome 6AL showing the physical location of *RgM4G52*. **(A)** Linkage map of *RgM4G52*; **(B)** physical interval (blue) where the four KASP markers are linked to *RgM4G52* anchored in Chinese Spring, with orange dots representing centromeres and dotted lines indicating the physical positions of each marker; **(C)** physical intervals anchored by markers linked to *RgM4G52* in *T. boeoticum.* Collinearity relationship of candidate genes in the *RgM4G52* gene mapping interval between *T. aestivum* Chinese Spring and *T. boeoticum* TA299. **(D)** Genetic linkage map of *RgM4G52*; **(E, F)** physical mapping of candidate genes in Chinese Spring and TA299.

There were 59 of the 171 clustered SNPs on 6AL 555–560 Mb (*p<1^e-8^* and |AFD|>0.6) chosen for the development of KASP markers. Four of them were successfully converted into KASP markers (*KASP-7*, *KASP-26*, *KASP-58*, *KASP-59*) (**Table 2**) and scored reliably on the parents as well as the red and green bulks (**Table 3**). These KASP markers were subsequently used to genotype 64 F_2_ plants derived from a cross between green-type G52 and red-type Z2921. Linkage analysis indicated that *KASP-58* was mapped 2.73 cM distal and that *KASP-26* was located 0.67 cM proximal to *RgM4G52* (**Figures 3A–C**).

**Table 2:** Primer sequences of KASP markers used for genetic mapping of *RgM4G52*.

Marker	Physical position (bp)	Allele 1 primer^a^	Allele 2 primer^b^	Common/reverse primer
*KASP-7*	556979833	GGGATTGGGGGAGCAGAGCA	GGGATTGGGGGAGCAGAGCG	GAGACGTCCTGTTGACTCCT
*KASP-26*	557091256	ACGTTATTCATACCAGAGCGTT	ACGTTATTCATACCAGAGCGTG	TGGAGGGAAAGGATGACACT
*KASP-58*	558796229	TGTGGACACCTTCAAGATGATC	TGTGGACACCTTCAAGATGATT	CTTCTTGCACTTGCCTTCCG
*KASP-59*	559401024	AGATCGAGCACGCCACGA	AGATCGAGCACGCCACGG	TCACTCCTCTCGTCCTTCCC

a A1 primer labeled with FAM: GAAGGTGACCAAGTTCATGCT.

b A2 primer labeled with HEX: GAAGGTCGGAGTCAACGGATT.

**Table 3:** Genotyping of Z2921 and G52 using KASP markers linked to *RgM4G52*.

Parents	Marker­genotype^a^			
	*KASP-7*	*KASP-26*	*KASP-58*	*KASP-59*
Z2921	AA	TT	CC	AA
G52	GG	GG	TT	GG

^a^ AA, CC, GG, and TT represent the haplotype results of SNP genotyping.

### Gene analysis of the *RgM4G52* genomic region

The sequences of the closely linked markers *KASP-26* and *KASP-58* were subjected to BLAST searches against the genome of Chinese Spring and that of *T. boeoticum* TA299 to determine their physical positions. *RgM4G52* was physically mapped to a 1.71-Mb region between the 557.09-Mb and 558.97-Mb regions of the Chinese Spring 6AL chromosome (IWGSC RefSeq v1.1) and between the 580.72-Mb and 582.33-Mb regions (1.61 Mb) of the TA299 6A^b^L chromosome (*T. boeoticum*) (**Figures 3A–C**). There were 40 and 42 predicted genes in the target physical regions in Chinese Spring and *T. boeoticum* TA299, respectively (IWGSC RefSeq v1.1; *T. boeoticum* TA299, **Supplementary Tables S1, S2**). In the Chinese Spring genome, seven genes may be associated with the anthocyanin biosynthesis pathway, including four cytochrome P450 family protein-related genes (Tanaka et al., 2009; Yang et al., 2004) (*TraesCS6A02G509500LC*, *TraesCS6A02G323900*, *TraesCS6A02G324000*, *TraesCS6A02G510200LC*), one universal stress protein family gene (Song et al., 2023a, 2023b; Gonzalez et al., 2008; Baudry et al., 2004; Stracke et al., 2010). (*TraesCS6A02G323800*), one peroxidase gene (*TraesCS6A02G324200*) (Grommeck and Markakis, 1964; Zapata et al., 1995; Kader et al., 2002; Zhang et al., 2005), and one F-box family protein-encoding gene (Zhang et al., 2017; Feder et al., 2015) (*TraesCS6A02G324300*). Four genes, namely, one cytochrome P450 protein-related gene (*Tm.TA299.r1.6AG0120810*), one peroxidase gene (*Tm.TA299.r1.6AG0120940*), a universal stress protein family gene, *Tm.TA299.r1.6AG0120790*, and an F-box family protein-encoding gene, *Tm.TA299.r1.6AG0121100LC*, were found in the *T. boeoticum* (TA299) genome and had a good collinear relationship with those of Chinese Spring (**Figures 3D–F**).

### Phenotypic and cytological molecular characterization of the amphiploid Syn-ABA^b^-34

**Figure 4:**
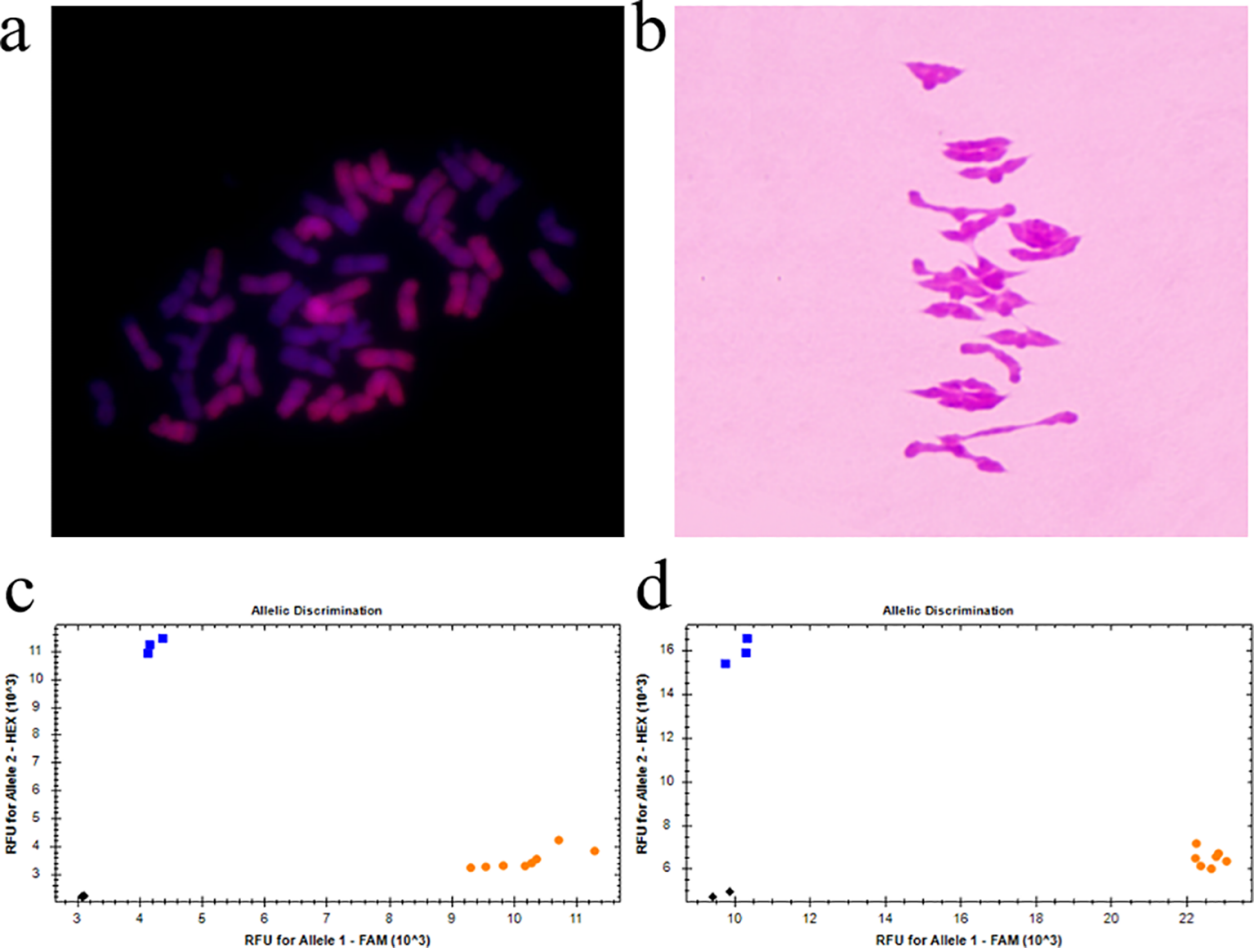
Cytological observations of the *T. dicoccum–T. boeoticum* amphiploid Syn-ABA^b^-34 and molecular detection using KASP markers linked to *RgM4G52*. **(A)** GISH identification of Syn-ABA^b^-34 using G52 genomic DNA as a probe; **(B)** chromosome pairing of PMCs in Syn-ABA^b^-34 with 21 bivalents; **(C)** molecular marker detection of Syn-ABA^b^-34 and its parents G52 and Z2921 using the KASP marker *KASP-26*, which is linked to *RgM4G52*. G52 in blue, Z2921 and Syn-ABA^b^-34 in yellow; **(D)** molecular marker detection of Syn-ABA^b^-34 and its parents G52 and Z2921 using the KASP marker *KASP-58* linked to *RgM4G52*. G52, blue; Z2921 and Syn-ABA^b^-34, yellow.

**Figure 5:**
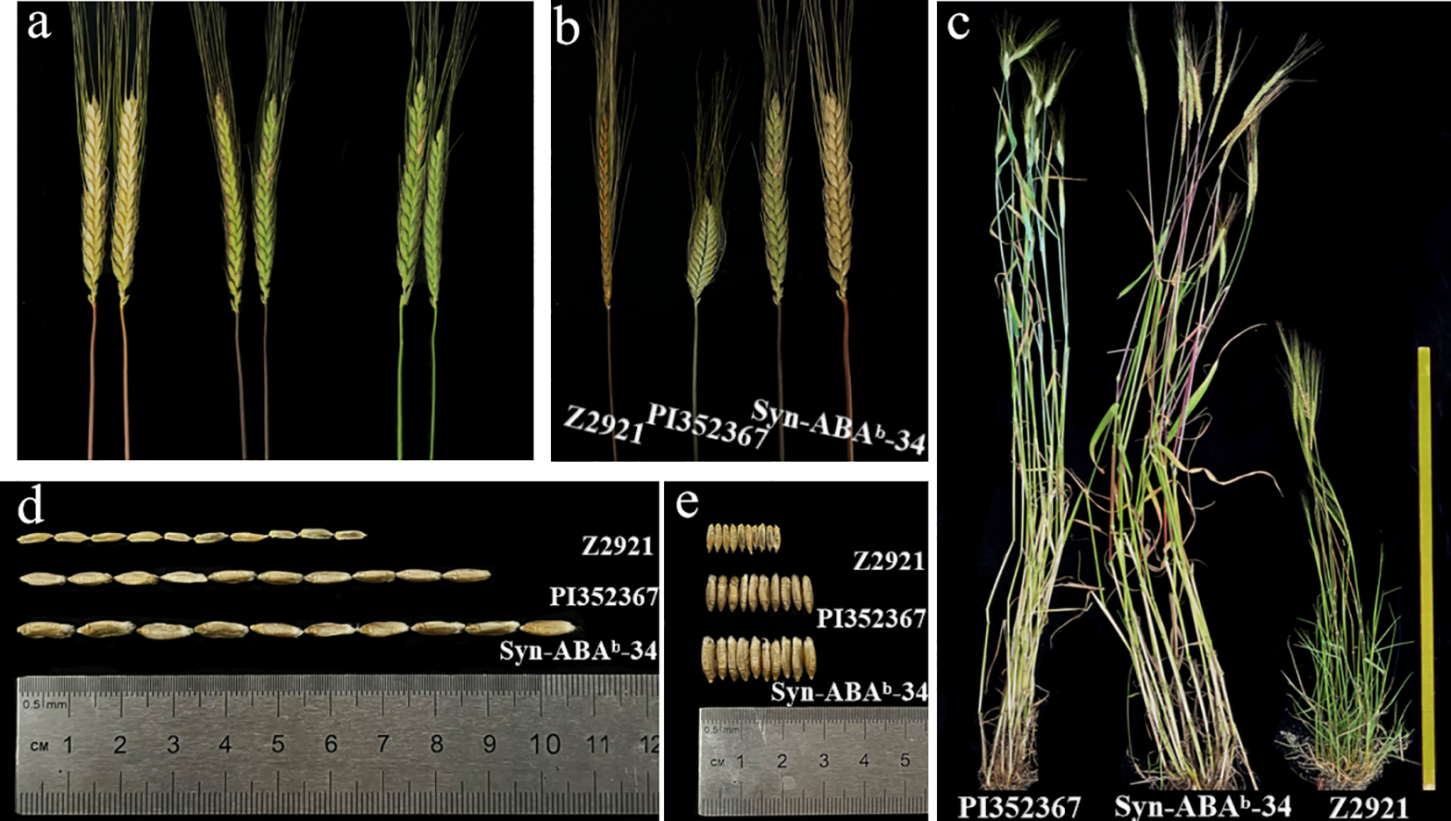
Phenotype of the *T. dicoccum–T. boeoticum* amphiploid Syn-ABA^b^-34 and its parents PI 352367 and Z2921. **(A)** The coloration of different stems from *T. dicoccum–T. boeoticum* amphiploid Syn-ABA^b^-34; **(B)** the stem coloration of Syn-ABA^b^-34 and its parents Z2921 and PI352367; **(C)** the plants of Syn-ABA^b^-34 and its parents Z2921 and PI352367; **(D)** the seed length of Syn-ABA^b^-34 and its parents Z2921 and PI352367; **(E)** the seed width of Syn-ABA^b^-34 and its parents Z2921 and PI352367.

An investigation of the number of chromosomes in the root tip showed that seven plants had 42 chromosomes and 2 plants had 41 chromosomes in the nine plants tested from amphiploid Syn-ABA^b^-34. Plants with 42 chromosomes were used for mc-GISH identification and chromosome pairing observation. Mc-GISH revealed 28 A-genome chromosomes among the amphiploid Syn-ABA^b^-34 chromosomes in the A-genome of Z5471 (**Figure 4A**). Chromosome pairing in PMCs (observed PMCs >50) at meiotic metaphase I was 2.58 rod II+17.92 ring II+0.16 I+0.16 III+0.08 IV in these plants with 42 chromosomes (**Figure 4B**), indicating the cytological stability of amphiploid Syn-ABA^b^-34. The amphiploid Syn-ABA^b^-34 was detected using the KASP markers *KASP-26* and *KASP-58* linked to *RgM4G52*; the results indicated that Syn-ABAb-34 carried the gene *RgM4G5*2 (**Figures 4C, D**). Field evaluation revealed that the color of stem changes from green to purple over time and finally changes from purple to red. Syn-ABA^b^-34 exhibited stem pigment accumulation similar to the diploid parent Z2921, whereas no pigment accumulation was detected in the tetraploid parent PI352367 (**Figures 5A–C**). Additionally, it was observed that the average length and width of 10 grains of Syn-ABA^b^-34 were greater than those of the parents Z2921 and PI352367 (**Figures 5D, E**).

## Discussion

Anthocyanins cause pigmentation in plant tissues and enhance plant resistance to biotic and abiotic stresses (Laikova et al., 2005). To date, there have been some reports of genes associated with pigmentation on leaves, glume shells, culms, seeds, etc., in wheat. *Rg* genes control glume shell color in diploid, tetraploid, and hexaploid wheat and are located on chromosomes 1A, 1B, 1D, and 2A (Khlestkina et al., 2010; McIntosh et al., 2013). Among these genes, *Rg-A1b* and *Rg-A1c* control the red and black (dark brown) glumes of diploid, tetraploid, and hexaploid wheat, respectively (Blanco et al., 1998; Salina et al., 2006) whereas *Rg-D1b* and *Rg-D1c* control the red (brown) and gray glumes, respectively, of hexaploid wheat (Khlestkina and Salina, 2006, 2009b, 2002a, 2002b). Three homoeologous genes for purple stems (*Pc-A1*, *Pc-B1*, *Pc-D1*), three homoeologs for purple leaf sheaths (*Pls-A1*, *Pls-B1*, *Pls-D1*), and three homoeologs for purple leaf blades (*Plb-A1*, *Plb-B1*, *Plb-D1*) have been mapped in close linkage with the red coleoptile genes *Rc-A1*, *Rc-B1*, and *Rc-D1* in wheat (Khlestkina et al., 2009a, 2010). Two genes responsible for purple anthers (*Pan-A1* and *Pan-D1*) have been mapped on chromosomes 7A (Blanco et al., 1998) and 7D (Khlestkina et al., 2009a) at short distances from *Rc-A1* and *Rc-D1*, respectively.

However, there have only been a few reports of pigmentation in glumes, culms, and rachides being simultaneously controlled by a single gene. In this study, a new pigmentation gene, *RgM4G52*, conferring red glumes, stems, and rachides, was identified in the *T. boeoticum* mutant Z2921 and mapped on chromosome arm 6AL flanked by the markers *KASP-26* and *KASP-58* within a 3.40-cM genetic interval corresponding to a 1.71-Mb physical region in the Chinese Spring genome (IWGSC RefSeq v1.1).

*RgM4G52* was physically mapped to a 1.61-Mb region between 580.72 Mb and 582.33 Mb on the TA299 6A^b^L chromosome arm (*T. boeoticum* TA299) (**Figures 3A–C**). Based on the gene functional annotation, there were four protein-coding genes, *Tm.TA299.r1.6AG0120790*, *Tm.TA299.r1.6AG0120810*, *Tm.TA299.r1.6AG0120940*, and *Tm.TA299.r1.6AG0121100LC*, in the target physical regions of the *T. boeoticum* genome (**Supplementary Tables S1, S2**; **Figures 3D, E**).

*Tm.TA299.r1.6AG0120790* was not annotated, and the homologous gene of *Tm.TA299.r1.6AG0120790* in Chinese Spring was *TraesCS6A02G323800*. Its functional annotation was that of a universal stress protein family gene. The universal stress protein family is involved in UV-B-induced flavonoid biosynthesis, and *VcUSP*s are coexpressed mainly with transcription factors from the MYB, AP2, zinc finger, and bHLH families (Song et al., 2023a, 2023b; Gonzalez et al., 2008; Baudry et al., 2004; Stracke et al., 2010). *Tm.TA299.r1.6AG0121100LC* was not annotated, and the homologous gene of *Tm.TA299.r1.6AG0121100LC* in the Chinese Spring is *TraesCS6A02G324300*. Its functional annotation was that of an F-box family protein. Members of the F-box serve as crucial negative regulators by mediating *CHS* and *PAL* degradation, which coordinately controls flavonoid biosynthesis (Zhang et al., 2017; Feder et al., 2015).

*Tm.TA299.r1.6AG0120810* was annotated as a desmethyl-deoxy-podophyllotoxin synthase, and the homologous gene of *Tm.TA299.r1.6AG0120810* in Chinese Spring was *TraesCS6A02G323900*. Its functional annotation was that of a cytochrome P450 protein-related gene. The enzymes flavonoid 3′-hydroxylase (F3′H) and flavonoid 3′,5′-hydroxylase (F3′5′H) play important roles in the anthocyanin biosynthesis pathway, and the genes encoding flavonoid 3′5′-hydroxylase (F3′5′h) and flavonoid 3′-hydroxylase (F3′h) belong to the cytochrome P450 monooxygenase gene family (Tanaka et al., 2009). There are no data on the cloning and/or mapping of these genes in wheat, except regarding one partial nucleotide sequence, F3′5′h (Yang et al., 2004). In the present study, *Tm.TA299.r1.6AG0120940* was annotated as a peroxidase gene, and the gene homologous to *Tm.TA299.r1.6AG0120940* in Chinese Spring was *TraesCS6A02G324200*. Its functional annotation was the same as that for *Tm.TA299.r1.6AG0120940*. All flavonoids have shown great binding affinity to peroxidase, and peroxidase can be degraded by anthocyanins as direct crop plant substrates or in the presence of H_2_O_2_ with anthocyanidins as substrates to oxidize and decolorize the anthocyanidins. The activity of peroxidase is negatively correlated with that of anthocyanins (Grommeck and Markakis, 1964; Zapata et al., 1995; Kader et al., 2002; Zhang et al., 2005). Now, the work is ongoing to clone and sequence these four genes, namely, *Tm.TA299.r1.6AG0120790*, *Tm.TA299.r1.6AG0120810*, *Tm.TA299.r1.6AG0120940*, and *Tm.TA299.r1.6AG0121100LC*. Then, candidate genes will be screened by sequence alignment. Finally, the function of candidate genes will be verified by the transgenic technique.

The flanking markers *KASP-26* and *KASP-58* developed in this study could be used as molecular markers to screen recombinant heterozygous plants, construct secondary F_2_ populations and develop markers, and further narrow the location interval to finely map and clone *RgM4G52*. There have been rare reports of pigmentation in glumes, culms, and rachides being simultaneously controlled by a single gene. *RgM4G52* may be a new recessive pigmentation gene. According to previous reports on the relationship between anthocyanin accumulation and plants, the red pigment accumulation in wheat with *RgM4G52* may be related to their adaptation to environmental stress conditions. It may be used as a morphological marker to assist in breeding and research related to gene functions and pigment synthesis pathways. Furthermore, it can also be used as a landscape crop for agro-ecological popular science tourism.

The expression of superior genes has been found to decrease or be completely inhibited when foreign genes have been transferred from a lower ploidy level (Ma et al., 1997; Ahmed et al., 2014). In this study, the amphiploid Syn-ABA^b^-34 had red stems at the jointing stage but had green glumes and rachides, indicating that the expression of *RgM4G52* was suppressed in the glumes and rachides. According to Hao et al. (2019), the amphiploid Syn-ABA^b^-34 can serve as a bridge to hybridize with elite wheat varieties, transferring this trait to common wheat and providing new germplasm resources for wheat breeding. Combined with molecular marker-assisted selection, the transfer of *RgM4G52* from diploid wheat to common wheat cultivars are ongoing using the amphiploid Syn-ABA^b^-34 as a bridge. The breeding of new wheat cultivars with red glumes/stems/rachides will provide new materials for the breeding of widely adaptable wheat varieties.

## Data Availability

The datasets presented in this study can be found in online repositories. The names of the repository/repositories and accession number(s) can be found in the article/**Supplementary Material**.
